# Appropriateness of DOAC Prescribing Before and During Hospital Admission and Analysis of Determinants for Inappropriate Prescribing

**DOI:** 10.3389/fphar.2018.01220

**Published:** 2018-10-30

**Authors:** Souad Moudallel, Stephane Steurbaut, Pieter Cornu, Alain Dupont

**Affiliations:** Centre for Pharmaceutical Research, Vrije Universiteit Brussel, Jette, Belgium

**Keywords:** direct oral anticoagulant (DOAC), inappropriate prescribing, risk factors, summary of product characteristics (SmPC), EHRA practical guidelines

## Abstract

**Background and Objectives:** Appropriate dosing of direct oral anticoagulants (DOACs) is required to avoid under- and overdosing that may precipitate strokes or thromboembolic events and bleedings, respectively. Our objective was to analyze the appropriateness of DOAC dosing according to the summaries of product characteristics (SmPC). Furthermore, determinants for inappropriate prescribing were investigated.

**Methodology:** Retrospective cohort study of hospitalized patients aged ≥60 years with at least one DOAC intake during hospital stay. Descriptive analyses were used to summarize the characteristics of the study population. Chi-square test was used to evaluate differences between DOACs. Binary logistic regression analysis was performed to assess determinants for inappropriate prescribing.

**Results:** For the 772 included patients, inappropriate dosing occurred in 25.0% of hospitalizations with 23.4, 21.9, and 29.7% for dabigatran, rivaroxaban, and apixaban, respectively (*p* = 0.084). Underdosing was most prevalent for apixaban (24.5%) compared to dabigatran (14.0%) and rivaroxaban (12.8%), *p* < 0.001. In 67.1% (apixaban), 26.7% (dabigatran), and 51.2% (rivaroxaban) of underdosed DOAC cases according to the SmPC, the dose would be considered appropriate according to the European Heart Rhytm Association (EHRA) guidelines. Overdosing was observed in 4.5% (apixaban), 4.7% (dabigatran), and 7.7% (rivaroxaban) of patients. For all DOACs, our analysis showed an age ≥80 years (*p* = 0.036), use of apixaban (*p* = 0.026), DOAC use before hospitalization (*p* = 0.001), intermediate renal function (*p* = 0.014), and use of narcotic analgesics (*p* = 0.019) to be associated with a higher rate of inappropriate prescribing. Undergoing surgery was associated with a lower odds of inappropriate prescribing (*p* = 0.012). For rivaroxaban, use of medication for hypothyroidism (*p* = 0.027) and the reduced dose (*p* < 0.001) were determinants for inappropriate prescribing. Treatment of venous thromboembolism was associated with less errors (*p* = 0.002). For apixaban, severe renal insufficiency (*p* < 0.001) and initiation in hospital (*p* = 0.016) were associated with less and the reduced dose (*p* < 0.001) with more inappropriate prescribing. No determinants were found in the dabigatran subgroup.

**Conclusions:** Inappropriate DOAC prescribing is frequent with underdosing being the most common drug related problem when using the SmPC as reference. More appropriate prescriptions were found when taking the EHRA guidelines into account. Analysis of determinants of inappropriate prescribing yielded insights in the risk factors associated with inappropriate DOAC prescriptions.

## Introduction

For the past 50 years, vitamin K antagonists (VKAs) have been the drugs of choice for long-term anticoagulation in patients with atrial fibrillation (AF) and venous thromboembolism (VTE). VKA therapy has been shown to be much more effective than aspirin or other antiplatelet drugs in reducing the risk of stroke and mortality in patients with AF, but is associated with well-documented problems including an increased risk of bleeding. According to the meta-analysis performed by Hart and colleagues, adjusted-dose warfarin reduced stroke by 64% (Hart et al., 2007). However, VKAs have a small therapeutic window, unfavorable pharmacokinetics and are very susceptible to drug-drug and nutritional interactions (Kirchhof et al., 2016). In addition, the anticoagulation effect is achieved only after 2–3 days and frequent international normalized ratio (INR) monitoring is required. These limitations stimulated the development of new alternative oral anticoagulant drugs known as the direct oral anticoagulants (DOACs).

The introduction of the DOACs widened the options for anticoagulation in AF and VTE. According to the pivotal trials, they have a superior safety profile with fewer major bleeding episodes compared to VKAs for both acute as well as extended treatment of patients with VTE (Ezekowitz et al., 2009; Schulman et al., 2009; Landman and Gans, 2011; Agnelli et al., 2013; Hess et al., 2013; Goodman et al., 2014). In AF, less intracranial bleedings and hemorrhagic strokes were documented compared to the VKAs (Barra et al., 2016; Lutz et al., 2017). Data from postmarketing surveillance studies provide assurance that the risks associated with their use are manageable and in line with the results seen in the phase III trials (Villines and Peacock, 2016). Several guidelines, including those of the European Society of Cardiology (ESC) and the European Heart Rhythm Association (EHRA), now recommend DOACs in preference to VKAs if anticoagulation is required in patients with non-valvular AF (Steffel et al., 2018).

DOACs have simplified and rather fixed dosing regimens and do not necessitate routine monitoring of their pharmacodynamic activity in contrast to the VKAs. However, each DOAC has a different dosing schedule and dose adaptations, mostly reductions, depend on one or more patient-specific factors including age, weight, renal function, serum creatinine, indication and concomitant medications. This increases the risk of dosing errors (Lutz et al., 2017; Whitworth et al., 2017). Although DOACs have provided possible solutions to several challenges associated with VKA therapy, there are also important drug-drug interactions to consider.

Even if DOACs have made anticoagulation more convenient, caution is warranted, especially in patients with renal insufficiency in order to decrease the risk of bleeding, stroke and VTE. All four currently European marketed DOACs (dabigatran, rivaroxaban, apixaban, and edoxaban) undergo renal excretion to some extent. The renal excretion of dabigatran accounts for 80% of the total plasma clearance, whereas smaller fractions of edoxaban (50%), rivaroxaban (35%), and apixaban (27%) are eliminated unchanged in the urine (Bayer, 2016; Boehringer-Ingelheim, 2016; Bristol-Myers Squibb/Pfizer, 2016; Daiichi-Sankyo, 2018). The advantages of DOACs compared to VKAs seem to decline with increasing renal impairement (Lutz et al., 2017). In patients with decreased renal function, DOACs can accumulate and dose adaptations are recommended. Administering DOACs in inappropriately adjusted drug dosages in renal patients is a medication error that is associated with adverse outcomes. Several studies have reported a poor adherence rate with dosing guidelines for the DOACs regardless of the renal function. These data suggest that inappropriate DOAC dosing is common and varies from 12.8 to 34.0% of adult hospitalized AF patients as well as other patients taking a DOAC (Armbruster et al., 2014; Larock et al., 2014; Kucey et al., 2016; Pattullo et al., 2016; Steinberg et al., 2016; Basaran et al., 2017; Howard et al., 2017; Shrestha et al., 2017; Whitworth et al., 2017).

The purpose of this observational study was to determine the prescribing accuracy of dabigatran etexilate, rivaroxaban, and apixaban, the three DOACs available in Belgium at the time of the study, according to their corresponding summary of product characteristics (SmPC) in a university hospital and to compare the results with earlier studies. We also compared the rate of inappropriate dosing resulting from DOAC prescriptions initiated before hospital admission with those initiated during hospitalization.

As there are few data in the literature on possible determinants of inappropriate prescribing, we further also explored potential determinants for inappropriate DOAC dosing. Identification of such factors can possibly lead to specific recommendations to improve DOAC prescription accuracy. We also aimed to document the incidence of bleeding complications and thromboembolic events following inappropriate dosing. Furthermore, as the primary analysis revealed that the majority of cases with inappropriate prescribing were due to underdosing according to the recommended doses in the SmPC, an additional analysis of the patients with underdosing was carried out taking into account the EHRA 2015 DOAC practical guidelines which differ from the SmPC, in particular regarding to dose adjustments (Heidbuchel et al., 2015).

## Methods

### Setting and study population

This retrospective cohort study was conducted on all hospitalized patients treated with either rivaroxaban, dabigatran, or apixaban (edoxaban was not yet available in Belgium during the study period) between 1 January 2016 and 31 December 2016 in the UZ Brussel. The UZ Brussel is a 721-bed university hospital located in the Brussels capital region. All patients aged ≥ 60 years having received at least one DOAC dose during their hospital stay were included. Patients undergoing dialysis were excluded.

### Data collection

DOAC prescriptions for hospitalized patients were queried from the hospital's medication claims database. Subsequently, patient data were retrieved from the electronic medical records (EMRs). In case a patient was hospitalized more than once in 2016, only the characteristics of the first admission were taken into account. We collected data on patient characteristics [age, sex, weight, body mass index (BMI), indication], prescribed DOAC including posology, baseline laboratory values, surgical procedures during hospital stay, department where the patient was admitted, co-medication use, in particular concomitant antiplatelet agents and concomitant P-glycoprotein (P-gp) and/or cytochrome P450 3A4 (CYP3A4) inhibitors and inducers, and whether or not the DOAC was already initiated before hospital admission (initiation setting). The renal function was estimated using the CG (Cockcroft and Gault) formula. The creatinine clearance (CrCl) was used to check appropriateness with regard to renal function as dose reductions in the SmPC are based on the CG formula. Information on recent hemoglobin levels and number of thrombocytes, bleeding history, and need for blood transfusions was also collected from the EMR. HAS-BLED and CHA_2_DS_2_-VASc scores were calculated for each admission (Mason et al., 2012; Zhu et al., 2015). An inappropriate DOAC dose was defined as a deviation of the drug-specific recommended dose as mentioned in the SmPC depending on renal function (CrCl/serum creatinine), age, weight and/or concomitant interacting medications (Appendix 1 in supplementary material). Underdosing and overdosing were respectively defined as administration of a lower or higher dose than recommended in the SmPC.

A prescription was deemed inappropriate in case of underdosing, overdosing, contra-indication, or when no clinical indication to initiate the DOAC was present or could be found in the EMR.

EMRs were additionally checked for clinical outcomes that might be related to inappropriate dosing, such as bleedings and thromboembolic events. Laboratory values were considered if they were measured in the interval of 3 days before medication initiation.

We used the 2015 version of the EHRA guidelines since these were in vigor when the DOACs were prescribed and because they only differ minimally with the updated 2018 guidelines.

### Ethics committee approval

The study was approved by the Medical Ethics Committee of the UZ Brussel with reference BUN 143201731174.

### Statistical analysis

Descriptive and statistical analyses were carried out with IBM SPSS Statistics version 25 (IBM Corp., Armonk, NY, USA). The Kolmogorov–Smirnov test was used to test the normality of the continuous variables. Histograms were also evaluated to assess normality. Mean with standard deviation and median with interquartile range (IQR) was used for normally and non-normally distributed variables, respectively. For categorical variables, frequencies were calculated. The Chi-square test was used to compare categorical variables. Continuous variables were compared with ANOVA. A significance level of 0.05 was used.

Binary logistic regression analysis was conducted to investigate risk factors for inappropriate DOAC dosing. Risk factors with a *p*-value < 0.1 in the univariable analysis were included in the multivariable model. Four models were constructed. First, all the DOACs were taken together after which each molecule was analyzed separately. For the logistic regression models, goodness of fit was assessed and residuals were reviewed. The odds ratios (OR) were reported with their 95% CI.

## Results

**Figure 1:**
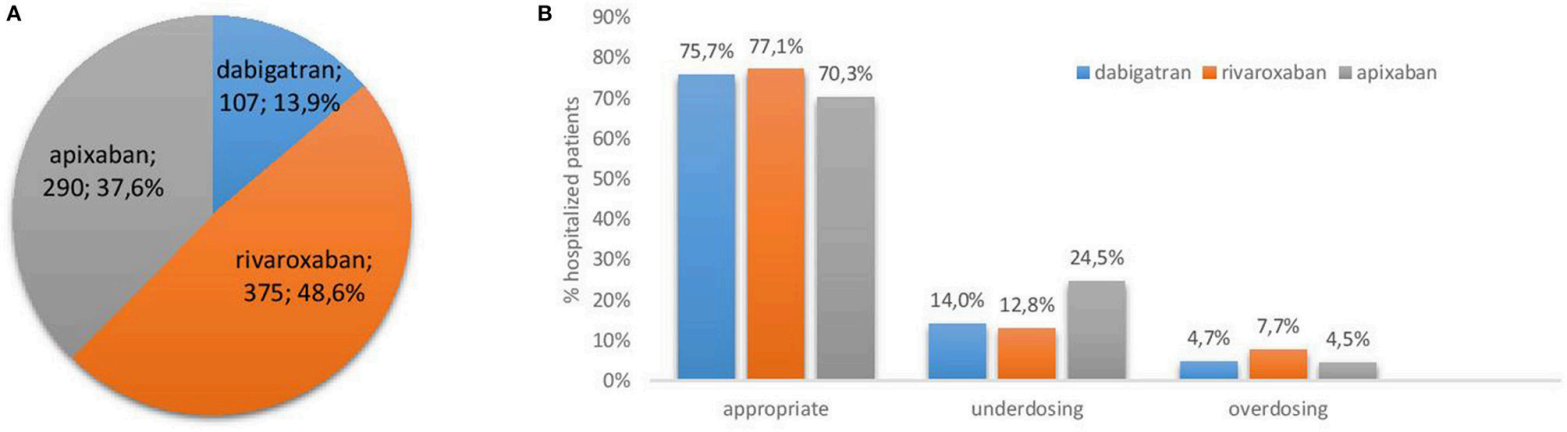
**(A)** Proportion of admissions where a specific DOAC was administered; **(B)** Appropriate vs. inappropriate (under-and overdosing) prescribing rates.

A total of 772 unique patients were included in this study. The patient characteristics are listed in Table 1. Figure 1A shows that rivaroxaban (375 patients; 48.6%) was the most frequently prescribed DOAC in the UZ Brussel in 2016, followed by apixaban (290 patients; 37.6%) and dabigatran (107 patients; 13.9%). Approximately 50% of the patients in all three DOAC groups, had a CrCl ≤ 60 mL/min as calculated with the CG formula. Values below 50 mL/min were more frequently observed in the apixaban group. The median renal function clearance of apixaban users (57 mL/min) was significantly lower than those treated with dabigatran or rivaroxaban (64 and 60 mL/min) (*p* < 0.001). Use of dabigatran in patients with severe renal insufficiency (CrCl < 30 mL/min) is contraindicated and occurred in 5 (4.7%) of dabigatran users. In four of these patients dabigatran was initiated before hospital admission. No other cases of contraindication were observed for dabigatran, rivaroxaban, or apixaban. Significantly more men were prescribed apixaban and rivaroxaban compared to dabigatran (*p* = 0.001). Prevention of stroke in AF was by far the most prevalent indication for prescribing a DOAC, and in the case of apixaban in up to 96.9% of admissions. In all groups, more than half of the AF patients showed a high risk for stroke (CHA_2_DS_2_-VASc ≥4). Rivaroxaban, dabigatran and to a lesser extent apixaban were in some cases also used for the treatment or secondary prevention of deep vein thrombosis or pulmonary embolism.

**Table 1:** Demographic data and specific characteristics of included patients.

	**Dabigatran**	**Rivaroxaban**	**Apixaban**	***p*-value**
	**(*n* = 107)**	**(*n* = 375)**	**(*n* = 290)**
Age in years, mean (± SD)	77.3.6 ± 8.7	77.9 ± 8.7	79.1 ± 8.6	0.011
Male gender, *n* (%)	44 (41.1)	196 (52.3)	143 (49.3)	0.001
Weight in kg, median (IQR)	75.0 (46.8–154.0)	75.0 (42.0–145.0)	73.0 (38.0–148.4)	0.068
BMI in kg/m^2^, median^*^ (IQR)	26.9 (18.3-41.0)	26.5 (15.4-58.0)	25.6 (13.0-51.4)	0.19
- BMI ≤ 18, *n* (%)	0 (0)	5 (1.3)	11 (3.8)
- BMI ≥ 30, *n* (%)	26 (24.3)	84 (22.4)	64 (22.1)
Duration of DOAC administration in hospital in days, median	4 (1–113)	5 (1–63)	4 (1–90)	0.318
Renal function (CG) in mL/min, median (IQR)	64 (21–177)	60 (13–345)	57 (12–165)	<0.001
- CrCl ≥ 60 mL/min (%)	50.5	47.7	44.8
- CrCl 50–59 mL/min (%)	21.5	17.3	11.4
- CrCl 40–49 mL/min (%)	10.3	14.5	18.6
- CrCl 30–39 mL/min (%)	6.5	8.0	13.5
- CrCl < 30 mL/min (%)	4.7	6.4	10.7
- Missing (%)	6.5	6.1	1.0
Length of stay in days, median (IQR)	10 (2–135)	9 (2–90)	10.5 (1–270)	0.05
Indication for DOAC (%)				<0.001
- Stroke prevention in AF	88.8	81.1	96.9
- Secondary VTE prevention	2.8	2.1	1.0
- Treatment of VTE	7.5	15.5	1.4
- Not specified	0.9	1.1	0.7
CHA_2_DS_2_-VASc, median (IQR)	4 (0–7)	4 (0–8)	4 (0–9)	/
- 0–1 (%)	8.4	5.6	3.1
- 2–3 (%)	24.3	36.5	27.9
- ≥4 (%)	67.3	52.5	66.9
HAS-BLED, median	2 (0–5)	3 (0–6)	3 (0–6)	/

*BMI, body mass index; DOAC, direct oral anticoagulant; CG, Cockcroft and Gault; AF, atrial fibrillation; VTE, venous thromboembolism*.

* *Missing renal function values were observed in 7, 23, and 3 patients for dabigatran, rivaroxaban and apixaban respectively due to a missing weight*.

### Prescribing accuracy of the DOACs

We identified 193 patients (25.0%) with inappropriate prescriptions when all three DOACs were considered together. Inappropriate prescriptions occurred in 23.4, 21.9, and 29.7% of patients receiving dabigatran, rivaroxaban, and apixaban, respectively (*p* = 0.084). As shown in Figure 1B, underdosing (17.4%) occurred more frequently than overdosing (6.1%) for all three DOACs and was most prominent in the apixaban group (24.5%) compared to dabigatran (14.0%) and rivaroxaban (12.8%) (*p* < 0.001). The prevalence of overdosing was lower for apixaban than for users of dabigatran and rivaroxaban (see Figure 1B; *p* < 0.001).

Factors contributing to inappropriate prescriptions are listed in Table 2. For apixaban the main factor leading to underdosing was an age ≥ 80 years without any additional factor justifying the use of the reduced dose (14.5%). Inappropriate dosing in the dabigatran and rivaroxaban group was mainly related to the patient's renal function (12.1 and 20.0% respectively). The reason for inappropriateness could not be evidenced for 13 cases in the apixaban group.

**Table 2:** Factors contributing to inappropriate prescriptions.

**Reason inappropriateness (*n*)**	**Dabigatran**	**Rivaroxaban**	**Apixaban**
	**(*n* = 107)**	**(*n* = 375)**	**(*n* = 290)**
Renal function, *n* (%) • Underdosing, *n* • Overdosing, *n*	13 (12.1) 10 3	75 (20.0) 48 27	3 (1.0) 1 2
Age, n (%) • Underdosing, *n* • Overdosing, *n*	2 (1.9) 0 2	/	42 (14.5) 42 0
Weight, n (%) • Underdosing, *n*	/	/	5 (1.7) 5
Age/weight (%) • Overdosing, *n*	/	/	9 (3.1) 9
Wrong frequency, n (%) • Underdosing, *n* • Overdosing, *n*	5 (4.7) 5 0	2 (0.5) 0 2	12 (4.1) 12 0
Contraindication, *n* (%)	4 (3.7)	0	0
No clinical indication, *n* (%)	1 (0.9)	5 (1.3)	2 (0.7)

### Bleeding complications and thromboembolic events

Prescribing physicians documented in respectively 2 and 10 admissions bleeding events for dabigatran and rivaroxaban in overdosed patients. In underdosed patients, two thromboembolic events were documented for apixaban users and one for rivaroxaban users.

### Medication reconciliation

**Figure 2:**
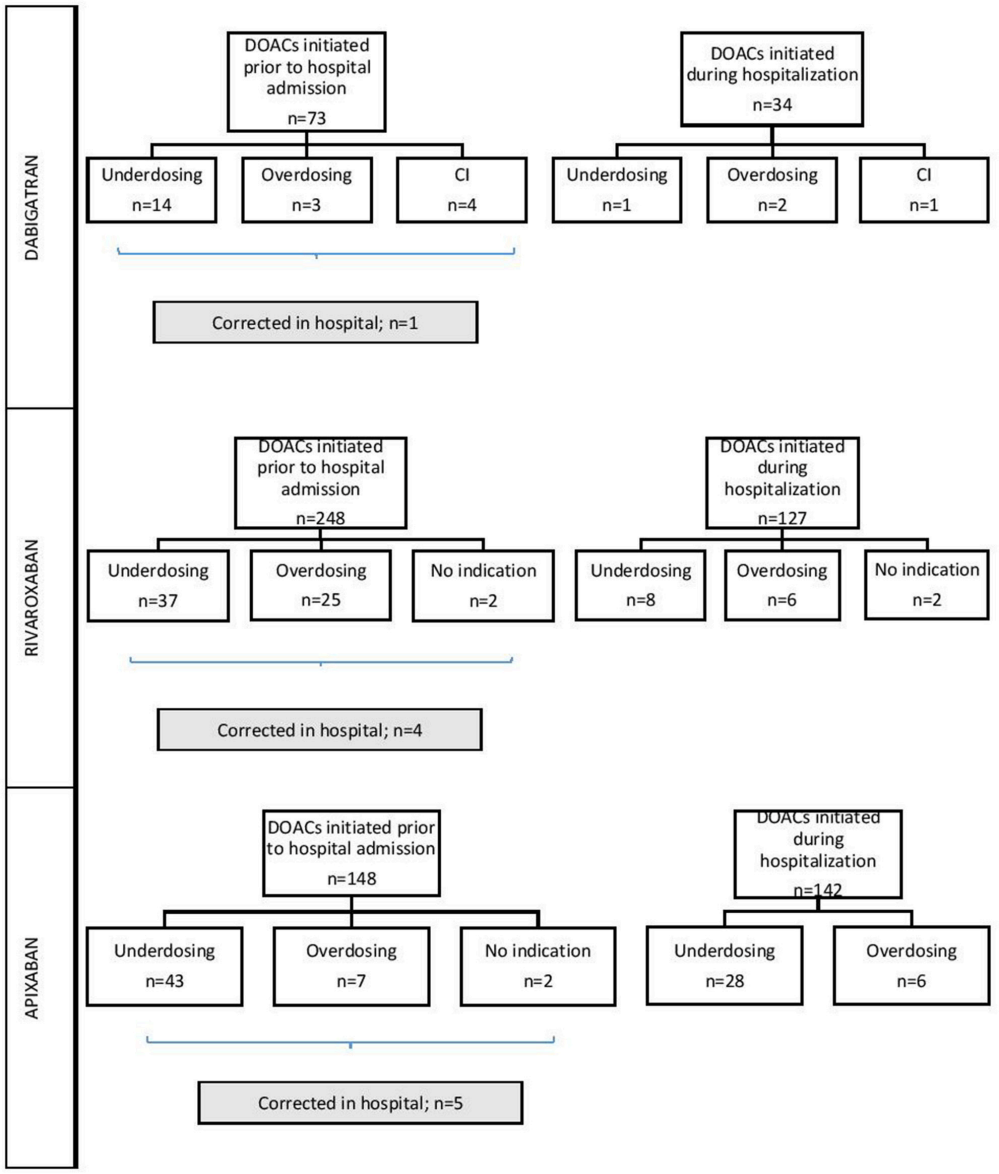
Inappropriate prescribing of dabigatran, rivaroxaban, and apixaban depending on the initiation setting. DOAC, direct oral anticoagulant; CI, contraindication.

When checking the appropriateness of prescriptions in patients where DOACs were already used prior to the hospitalization, we observed inconsistencies according to the SmPC in 28.8% (*n* = 21), 25.8% (*n* = 64), and 35.1% (*n* = 52) of dabigatran, rivaroxaban, and apixaban users, respectively. In only 1, 4, and 5 of these patients doses were corrected during hospitalization by physicians (see Figure 2). For the DOACs newly initiated during hospitalization and compared to those already prescribed before hospitalization, inappropriate prescription rates were observed in 11.8% (*n* = 4) of the patients with dabigatran (*p* = 0.049), 12.6% (*n* = 16) with rivaroxaban (*p* = 0.001) and 23.9% (*n* = 34) with apixaban (*p* = 0.037) (Figure 2). Of all inappropriate apixaban prescriptions, 39.5% were initiated in the UZ Brussel, whereas this was 16.0 and 20.0% for dabigatran and rivaroxaban, respectively.

### EHRA guidelines

In the apixaban group, 67.1% of the underdosed cases according to the SmPC would be considered appropriate when applying the EHRA guidelines. For dabigatran and rivaroxaban this accounts for 26.7 and 51.2%, respectively. The overall underdosing rate according to the EHRA guidelines is 7.0 vs. 17.4% according to the SmPC.

### Determinants for inappropriate prescribing: regression analysis

The results of the regression analysis are shown in Table 3. An age ≥ 80 years (*p* = 0.036; adj. OR 1.54; 95% CI 1.03–2.30), an intermediate renal function (CrCl 30–49 mL/min) (*p* = 0.014; adj. OR 1.74; 95% CI 1.12–2.71), and use of apixaban as DOAC (*p* = 0.026; adj. OR 1.61; 95% CI 1.06–2.44) were factors significantly associated with inappropriate prescribing when considering all DOACs together. Use of narcotic analgesics also appeared to be a risk factor for inappropriate prescribing (*p* = 0.019; adj. OR 1.69; 95% CI 1.09–2.60). The initiation of a DOAC in hospital was significantly associated with less inappropriate prescribing (*p* = 0.001; adj. OR 0.49; 95% CI 0.33–0.75). Also, when patients on DOACs were admitted to the hospital in the context of elective surgery, less inappropriate prescribing was observed (*p* = 0.012; adj. OR 0.50; 95% CI 0.29–0.86) (see Table 3).

**Table 3:** Multivariable logistic regression for the determination of risk factors that can lead to inappropriate prescribing.

	**Variable**	**Adjusted OR (95% CI)**
ALL DOACs	Age < 80 years (REF) ≥ 80 years	1.54 (1.03–2.30)
	Surgery Yes No (REF)	0.50 (0.29–0.86)
	DOAC Dabigatran Apixaban Rivaroxaban (REF)	1.15 (0.66–2.02) 1.61 (1.06–2.44)
	DOAC initiated before admission No Yes (REF)	0.49 (0.33–0.75)
	Renal function CG CrCl < 30 mL/min CrCl 30–49 mL/min CrCl ≥ 50 mL/min (REF)	0.76 (0.37–1.57) 1.74 (1.12–2.71)
	Use of narcotic analgesics Yes No (REF)	1.69 (1.09–2.60)
RIVAROXABAN	Dosage 15 mg 20 mg (REF)	4.41 (2.22–8.75)
	Indication Stroke prevention in AF (REF) Secondary VTE prevention Treatment of VTE	1.85 (0.25–13.73) 0.07 (0.01–0.39)
	Use of medication for hypothyroidism Yes No (REF)	2.30 (1.10–4.81)
APIXABAN	Dosage 2.5 mg 5 mg (REF)	14.03 (6.15–32.04)
	DOAC initiated before admission No Yes (REF)	0.43 (0.21–0.85)
	Renal function CG CrCl < 30 mL/min CrCl 30–49 mL/min CrCl ≥ 50 mL/min (REF)	0.06 (0.02–0.21) 0.97 (0.43–2.17)

*Only significant determinants are shown (p < 0.05). DOAC, direct oral anticoagulant; CG, Cockcroft and Gault; AF, atrial fibrillation; VTE, venous thromboembolism; REF, reference group*.

Use of the reduced rivaroxaban dose (15 mg) (*p* < 0.001; adj. OR 4.41; 95% CI 2.22–8.75) and use of medication for hypothyroidism (*p* = 0.027; adj. OR 2.30; 95% CI 1.10–4.81) were associated with a higher odds of inappropriate prescribing compared to the full dose. The indication for which rivaroxaban was used was also found to have an influence on the appropriateness of prescribing. Treatment of VTEs was associated with a lower odds of inappropriate prescribing compared to stroke prevention in AF (*p* = 0.002; adj. OR 0.07; 95% CI 0.01–0.39).

For apixaban, severe renal insufficiency showed a lower odds for inappropriate prescribing when compared to a normal renal function (*p* < 0.001; adj. OR 0.06; 95% CI 0.02–0.21). Also, when apixaban was prescribed for the first time during hospitalization, a lower odds for inappropriate prescribing was observed (*p* = 0.016; adj.0.43; 95% CI 0.21–0.85) compared to when apixaban was already initiated before admission.The use of the reduced dose (2.5 mg) was associated with a higher odds of inappropriate prescribing compared to the full dose of 5 mg (*p* < 0.001; adj. OR 14.03; 95% CI 6.15–32.04). The subgroup analysis for dabigatran revealed no risk factors for inadequate prescribing.

### Concomitant use of antiplatelet drugs

Concomitant administration of low dose acetylsalicylic acid or clopidogrel was observed in 28.9% of all patients. About a quarter (25.1%) of these antiplatelet drugs were newly initiated at the UZ Brussel. We found low hemoglobin values (women <12 g/dL; men <13 g/dL) in 61.0% of patients where a DOAC was given together with an antiplatelet drug, whereas a low hemoglobin value was observed in only 43.2% of patients where the prescription of a DOAC was not combined with an antiplatelet drug (*p* < 0.001). Combination with an antiplatelet drug was most often observed with rivaroxaban (48.0%), followed by apixaban (42.2%), and dabigatran (9.9%). The prevalence of patients on dual antiplatelet therapy (DAPT) in our study was 0.4, 1.3, and 1.0% for dabigatran, rivaroxaban, and apixaban, respectively. Hemoglobin values were low in the majority of these patients (66.7% in combination with dabigatran; 70.0% with rivaroxaban and 62.5% with apixaban), and were associated with at least 6 documented bleeding events including hematuria (*n* = 3), hemoptysis (*n* = 1), epistaxis (*n* = 1), and bleeding gums (*n* = 1).

## Discussion

### Inappropriate dosing

The main finding of this observational study was that DOACs are frequently dosed inappropriately in patients with AF or VTE despite the fact that they are in use for several years now, with underdosing being more common than overdosing. Inappropriate dosing rates in this study were found in the range reported in the literature for adult AF patients on DOACs (Kucey et al., 2016; Steinberg et al., 2016; Basaran et al., 2017; Shrestha et al., 2017). Inappropriate dosing rates in AF patients varied from 7.7 to 42.0% for dabigatran, from 13.0 to 29.8% for rivaroxaban, and from 12.7 to 48.1% for apixaban. Other studies, including a mix of AF and VTE patients, such as the present study, showed similar results (Armbruster et al., 2014; Larock et al., 2014; Pattullo et al., 2016; Howard et al., 2017; Whitworth et al., 2017). Dosing appropriateness in these studies was evaluated based on the SmPC or the medication appropriateness tool (MAI). In an Australian study, where 34% of the study population was prescribed a DOAC inappropriately, treatment was contraindicated in 40%, mainly due to severe renal impairment (Pattullo et al., 2016). In our study, an absolute contraindication was observed in 4.7% of the dabigatran users with a CrCl < 30 mL/min.

### Underdosing

We observed a high percentage of underdosing in the present study, in particular for apixaban, confirming earlier studies (Kucey et al., 2016; Pattullo et al., 2016; Steinberg et al., 2016; Basaran et al., 2017). This could be due to the relatively more complex dosing instructions for this DOAC, necessitating a dose reduction only when at least 2 out of the 3 following factors are met: serum creatinine ≥ 1.5 mg/dL, weight ≤ 60 kg, and/or age ≥ 80 years. This contrasts with both other DOACs where only one factor is sufficient to entail a dose adaptation.Our results are also in line with a recent report of the Belgian Health Care Knowledge Centre (KCE) indicating that a large proportion of Belgian patients (43.0%) treated with DOACs was found to receive a reduced dose (Van Brabandt et al., 2017). Moreover, in our study a once daily administration was prescribed in 4.5 and 5.6% of the patients for apixaban and dabigatran, respectively. This was also considered as underdosing since these two DOACs require a twice daily administration for each indication that is mentioned in the SmPC, with the exception of dabigatran used for VTE prevention after knee or hip replacement surgery. The high incidence of underdosing with apixaban seems clinically relevant as it was reported to beassociated with a nearly 5-fold increased stroke risk in AF patients (hazard ratio: 4.87; 95% CI: 1.30–18.26) (Yao et al., 2017).

In general, it is known that physicians tend to prescribe lower than recommended doses of anticoagulation because they fear bleeding events (Sen and Dahlberg, 2014). Fear of bleeding is a widely acknowledged reason for prescribing subtherapeutic doses or to refrain from anticoagulation initiation in high risk patients (Ding et al., 2017). This was also seen at discharge of our study population where anticoagulation was ceased in 19 AF patients. Documented reasons included high fall risk, high bleeding risk, and palliative setting. Although all anticoagulant therapies are associated with some degree of bleeding risk, this adverse event may be mitigated by consistently using evidence-based clinical evaluation scales. Such scales are designed to predict the bleeding risk during anticoagulant therapy or to help physicians identify AF patients who require anticoagulation therapy to reduce their stroke risk such as the HAS-BLED and CHA_2_DS_2_-VASc score, respectively (Sen and Dahlberg, 2014). In addition, in a recent study it was suggested that physicians are also moved by the prospect of harms more than by identically sized benefits (Avorn, 2018).

### Overdosing

Although overdosing occured less frequently than underdosing in the present study, it is also a clinically meaningful problem. Bleeding events after overdosing were reported in 2 patients on dabigatran and 10 on rivaroxaban who, interestingly, all had a CrCl ≤ 50 mL/min. Values below 30 mL/min were seen in 3 patients on rivaroxaban. It is known from the literature that overdosing due to the use of a standard DOAC dose in patients with severe renal impairment is associated with a doubled risk of bleeding while the effect on stroke reduction remains identical (Yao et al., 2017). Smythe et al. found that in patients with dabigatran related major bleeding, more than one-third had an excessive dose based on their renal function (Smythe et al., 2015).

### Correction of inappropriate prescriptions

For the three DOACs taken together, correction of inadequate prehospital doses after admission occurred in <10% of cases. This may indicate that once a DOAC is initiated in an inappropriate way, there is only a small chance that it will be corrected afterwards. Physicians make less dosing errors when newly starting a DOAC during hospital admission, but seem more prone to prescribe an inappropriate dose when continuing a DOAC that was already initiated before hospitalization. This might be due to insufficient consideration of patient characteristics that may have changed in the time between the initiation of the medication and the current hospitalization.

### EHRA guidelines vs. SmPC

In the present study, we used the SmPC as basis for the evaluation of prescribing accuracy because the SmPC is in our institution the basis for the local guidelines on dose selection. A few years ago, the EHRA published a practical guide for informing physicians on the use of the different DOACs in patients with non-valvular AF in clinical practice based on available evidence and expert opinion. The dosing recommendations in these guidelines, which were updated in 2013, 2015, 2016, and 2018 are less strict than those in the SmPCs which gives the physicians more freedom to adapt the dose for an individual taking into account patient specific factors (e.g., bleeding risk or concomitant antiplatelet use). The large number of underdosed DOAC prescriptions as based on the SmPCs observed in the present study, prompted us to do an additional analysis using the EHRA guidelines. We found that 7 out of 10 of underdosed apixaban prescriptions according to the SmPC, and 3 and 5 out of 10 for dabigatran and rivaroxaban respectively, would be considered appropriate according to the EHRA guidelines. We could only find one other study where the prevalence of inappropriate DOAC doses was evaluated using the EHRA guidelines (Ruiz Ortiz et al., 2018). This study revealed an inappropriate dosing rate of 32.0%. To our knowledge, our study is the first to compare appropriateness rates for underdosing according to the SmPC and EHRA guidelines.

### Determinants of inappropriate prescribing

Analysis of possible determinants revealed that the use of DOACs in hospitalized patients aged 80 years or more is associated with a higher odds for inappropriate prescribing compared to patients with an age <80 years. We would expect that age would also be a determinant for inappropriate prescribing for the individual DOACs, especially for dabigatran and apixaban where dose adaptations are made in function of the age, but we could not detect such relation possibly because of lack of power. Use of apixaban as DOAC was also associated with a higher odds of inappropriate prescribing compared to the use of rivaroxaban reinforcing the finding that it was the DOAC with the highest inappropriateness rate in our study.

Severe renal insufficiency (CrCl < 30 mL/min) was not associated with a higher odds for inappropriate prescribing, in contrast to moderate renal impairment, possibly because DOACs are prescribed more cautiously in the former patients. In addition, we assume that patients who were admitted to the hospital for elective surgery underwent an extensive medication check by the anesthetist resulting in less inappropriate prescribing of the DOACs in this population. An unexpected observation for all DOACs was that narcotic analgesics use was associated with a higher odds for inappropriate prescribing. The observation, for rivaroxaban, that the use of medication for hypothyroidism was associated with a higher odds for inappropriate prescribing was also unexpected.

Furthermore, the use of the reduced dose of 15 mg rivaroxaban was associated with a higher odds for inadequate prescribing compared to the full dose of 20 mg. This matches the observation that 40.4% of reduced rivaroxaban doses were manifestations of inappropriate dosing. This is also in line with the conclusions of the phase IV observational trial studying rivaroxaban use in AF patients (XANTUS) which revealed that 15% of patients with a good creatinine clearance received the reduced dose of 15 mg (Camm et al., 2016). The lower odds ratio for inappropriate prescribing of rivaroxaban for treatment of VTE may be related to the fact that the dose in this case is less dependent on renal function as compared to the indication AF and consequently less subject to errors. However, inappropriate prescribing in VTE treatment only occurred in 3.5% of all patients on rivaroxaban so the small number of observations requires cautious interpretation.

For the apixaban subgroup, our analysis showed a significantly lower odds for inappropriate prescribing when the CrCl was lower than 30 mL/min. Under these circumstances, the SmPC mentions that the reduced dose should be used whereas a dose reduction is only allowed when a serum creatinine concentration ≥ 1.5 mg/dL converges with a weight ≤ 60 kg and/or an age ≥ 80 years. In all patients where the reduced dose of 2.5 mg was administered twice daily, this was inappropriate in approximately half of the cases. In 42 admissions (14.5% of apixaban use), a reduced dose of apixaban was administered when only the age criterion was met (≥ 80 years). In 5 patients (1.7% of apixaban use), only the weight criterion (≤ 60 kg) was decisive to prescribe the reduced dose. This is in line with the observation that apixaban is dosed subtherapeutically in 24.5% of all prescriptions.

### DOACs combined with antiplatelet therapy

More frequent bleeding events were documented in the literature when antiplatelet therapy is prescribed concomitantly with oral anticoagulants (Walenga and Adiguzel, 2010; Sen and Dahlberg, 2014). Our data show that the combination of DOACs with antiplatelet drugs is quite common since it was observed in almost one third of all hospital admissions included in this study. This is similar to findings in the ORBIT registry in which 25% of the entire AF cohort received an anticoagulant in combination with antiplatelet therapy (Steinberg et al., 2016).

The high rate of concomitant antiplatelet drug use is also in line with the pivotal DOAC trials. The RCTs show that 30 to 40% of the participants used acetylsalicylic acid in combination with the DOAC.

In addition, combination of a DOAC with DAPT increases the bleeding risk as evidenced by three trials that compared DOACs with placebo in the context of acute coronary syndrome and DAPT (Alexander et al., 2011; Mega et al., 2012; Dans et al., 2013). In our study population, at least one in four DOAC patients receiving DAPT presented with a bleeding.

### Study limitations

This study has several limitations. First, it is a single center study limiting the generalizability of our results although they seem to be in line with other recently published data on inappropriate DOAC prescribing and underdosing in particular. Given the fact that this study is retrospective and not all clinical outcomes might have been registered, our results may be biased by inaccurate or incomplete information. Notably the number of bleedings as well as thromboembolic events detected in the study may be underestimated. Moreover, weight is a dynamic variable that may not have been updated in the medical charts. Of the studied DOACs, apixaban requires weight-dependent dose adaptations and hence is prone to inappropriate dosing when this parameter is not assessed or adapted at the time of DOAC initiation.

## Conclusion

An evaluation of the prescribing accuracy of DOACs in our hospital suggests prescribing patterns that are often inconsistent with the SmPC. Underdosing seems to occur more often, in particular in patients prescribed apixaban. The complexity of appropriate DOAC dosing, depending on aspects such as therapeutic indication, co-medication, renal function, and other patient related factors such as age and/or weight, as well as the setting of DOAC initiation (prehospital vs. hospital) contributes to prescribing errors. Underestimation of the necessity for dose reduction and insufficient attention for an impaired renal function may be explanations in case of overdosing whereas fear for bleeding and insufficient knowledge of the set of conditions requiring a dose reduction may lead to underdosing. The EHRA guidelines seem to be less strict than the SmPC and/or more pragmatic since a high proportion of the underdosed cases according to the SmPC were classified as conform with respect to the EHRA guidelines.

Integrating dosing algorithms in the hospital's computerized physician order entry system may overcome some of the problems associated with DOAC use through the generation of tailored dosing advices at the moment of prescription taking into account patient specific characteristics, diagnoses and laboratory results as well as co-medication. Medication reconciliation at admission and discharge in combination with medication review by clinical pharmacists may be another cornerstone to address inappropriate prescribing as the majority of incorrect DOAC doses found in our study resulted from the prehospital use of these drugs. Further, education of physicians with regard to adequate prescribing of DOACs is important and should receive more attention in order to avoid drug related problems.

## Author contributions

SM did the acquisition of the data and obtained ethical approval. All authors contributed to the conception and design of the study, and helped with the analysis and interpretation of the data. SM drafted the manuscript. All the authors revised the manuscript critically and approved the final manuscript.

### Conflict of interest statement

The authors declare that the research was conducted in the absence of any commercial or financial relationships that could be construed as a potential conflict of interest.
