# Supplementary material for: Appropriateness of DOAC Prescribing Before and During Hospital Admission and Analysis of Determinants for Inappropriate Prescribing
Source: Front Pharmacol. 2018 Oct 30;9:1220. doi: 10.3389/fphar.2018.01220 (PMC6218888; doi:10.3389/fphar.2018.01220)
Supplement: Supplementary file 1 [file Table_1.DOCX]

Appendix 1.

| **STROKE PREVENTION IN NON-VALVULAR ATRIAL FIBRILLATION** | | | | | |
| --- | --- | --- | --- | --- | --- |
| DOAC | CrCl (mL/min) | | | | |
|  | >50 | 30-50 | 15-29 | <15 | |
| Dabigatran° | 150 mg twice daily | 110 mg twice daily if high bleeding risk | Contraindicated | Contraindicated | |
| Rivaroxaban | 20 mg once daily | 15 mg once daily | 15 mg once daily | Not recommended | |
| Apixaban* | 5 mg twice daily | No adjustment needed | 2.5 mg twice daily | Not recommended | |
| **VTE TREATMENT OR SECONDARY PREVENTION** | | | |  | |
| DOAC | CrCl (mL/min) | | | | |
|  | >50 | 30-50 | <30 | <15 | |
| Dabigatran | 150 mg twice daily (after 5-10 days of parenteral anticoagulation) | 110 mg twice daily if high bleeding risk | Contraindicated | Contraindicated | |
| Rivaroxaban | 15 mg twice daily for 21 days | No adjustment needed | Use with caution | | Not recommended |
| Apixaban | **Treatment**: 10 mg twice daily for 7 days  **Prevention**: 2.5 mg twice daily | No adjustment needed | Use with caution | Not recommended | |

**Table S1: DOAC dosing recommendations according to the summary of product charecteristics (SmPC).**

°The SmPC strongly recommends the use of the reduced dose (110 mg twice daily) in patients ≥ 80 years or taking verapamil concomitantly

*Reduce to 2.5 mg twice daily if any two of the following are present: age ≥ 80 years, weight ≤ 60 kg, serum creatinine ≥1.5 mg/dL
